# Pif1 is essential for efficient replisome progression through lagging strand G-quadruplex DNA secondary structures

**DOI:** 10.1093/nar/gky1065

**Published:** 2018-11-05

**Authors:** Danielle Dahan, Ioannis Tsirkas, Daniel Dovrat, Melanie A Sparks, Saurabh P Singh, Roberto Galletto, Amir Aharoni

**Affiliations:** 1 Department of Life Sciences and the National Institute for Biotechnology in the Negev, Ben-Gurion University of the Negev, Be’er Sheva 84105, Israel; 2 Department of Biochemistry and Molecular Biophysics, Washington University School of Medicine, St. Louis, MO 63110, USA

## Abstract

Pif1 DNA helicase is a potent unwinder of G-quadruplex (G4) structures *in vitro* and functions to maintain genome stability at G4 sequences in *Saccharomyces cerevisiae*. Here, we developed and utilized a live-cell imaging approach to quantitatively measure the progression rates of single replication forks through different G4 containing sequences in individual yeast cells. We show that in the absence of Pif1, replication rates through specific lagging strand G4 sequences *in vivo* is significantly decreased. In contrast, we found that in the absence of Pif1, replication rates through the same G4s on the leading strand are not decreased relative to the respective WT strains, showing that Pif1 is essential only for efficient replication through lagging strand G4s. Additionally, we show that a canonical PIP sequence in Pif1 interacts with PCNA and that replication through G4 structures is significantly slower in the absence of this interaction *in vitro* and *in vivo*. Thus, Pif1–PCNA interaction is essential for optimal replisome progression through G4 sequences, highlighting the importance of coupling between Pif1 activity and replisome progression during yeast genome replication.

## INTRODUCTION

G-quadruplex (G4) structures are extremely stable non-canonical four-stranded DNA secondary structures formed by non-Watson-Crick base pairing. The structure is composed of stacks of four planar guanine bases held together by Hoogsteen-type hydrogen bonds (1). G4s are widely spread across both *Saccharomyces cerevisiae* and human genomes and many G4 motifs are evolutionary conserved (2–4). Their association with distinct genomic features, such as promoters and transcriptional regulatory sites (5), suggests that G4s have a functional role *in vivo*. However, due to their thermal stability, the formation of G4 structures can be harmful to processes like DNA replication. During DNA replication, G4s can act as a stable kinetic trap and influence fork progression (6). In eukaryotes multiple helicases have been identified with the ability to bind and/or unwind G4s *in vivo* (7), possibly with some level of functional redundancy. In humans, mutations in helicases that unwind G4s *in vitro* are associated with diseases that lead to genomic instability, highlighting the importance of G4 unwinding to prevent premature aging and cancer (8).

Pif1 is a highly conserved 5′-3′ helicase identified in nearly all eukaryotes and in some prokaryotes and viruses (9)*. Saccharomyces cerevisiae* Pif1 is present in both the nucleus and mitochondria, and has multiple roles in the cell, such as regulation of telomerase activity and Okazaki fragment maturation (10). Additionally, Pif1 is a particularly efficient unwinder of G4 structures *in vitro* and growing evidence suggest that Pif1 binds and resolves G4 structures *in vivo* to prevent genome instability (11–13). Whether the absence of Pif1 affects the progression of replication through G4s has been debated in recent studies. A previous study (12) showed a regional fork slow down around G4 motifs in the presence of hydroxyurea (HU) in Pif1-deficient cells. However, later studies did not detect any substantial arrest under these conditions (14,15). Despite the growing number of reports regarding Pif1’s significant role at G4s *in vivo*, it remains unclear if this role is associated with facilitating replication fork progression at G4 sites or if these sequences are resolved post replication. Moreover, to date, there is no quantitative information on the extent of replication fork slow down at G4 sites in the absence of Pif1.

The complex task of eukaryotic DNA replication is carried out by the replisome, a dynamic protein complex responsible for all genome duplication (16). An essential protein in the eukaryotic replisome is proliferating cell nuclear antigen (PCNA), a member of the conserved sliding clamp family. PCNA functions as a key coordinator of replication by orchestrating the activity of various regulatory and metabolic enzymes at replication sites by interacting with a conserved motif in its partners, termed PCNA interacting protein (PIP) box (17). Recently, Pif1 was shown to interact with PCNA through a non-canonical PIP box located at the C-terminus of Pif1 (18). However, Pif1 also contains two canonical PIP sequences, one at the C-terminus of the helicase domain and the other in the middle of the helicase domain, whose importance for PCNA interaction is still unclear. While Pif1–PCNA interaction was shown to be important for break-induced replication (BIR) in yeast (18), it is unknown whether Pif1–PCNA interaction is important for replication through G4 structures in yeast.

**Figure 1.**
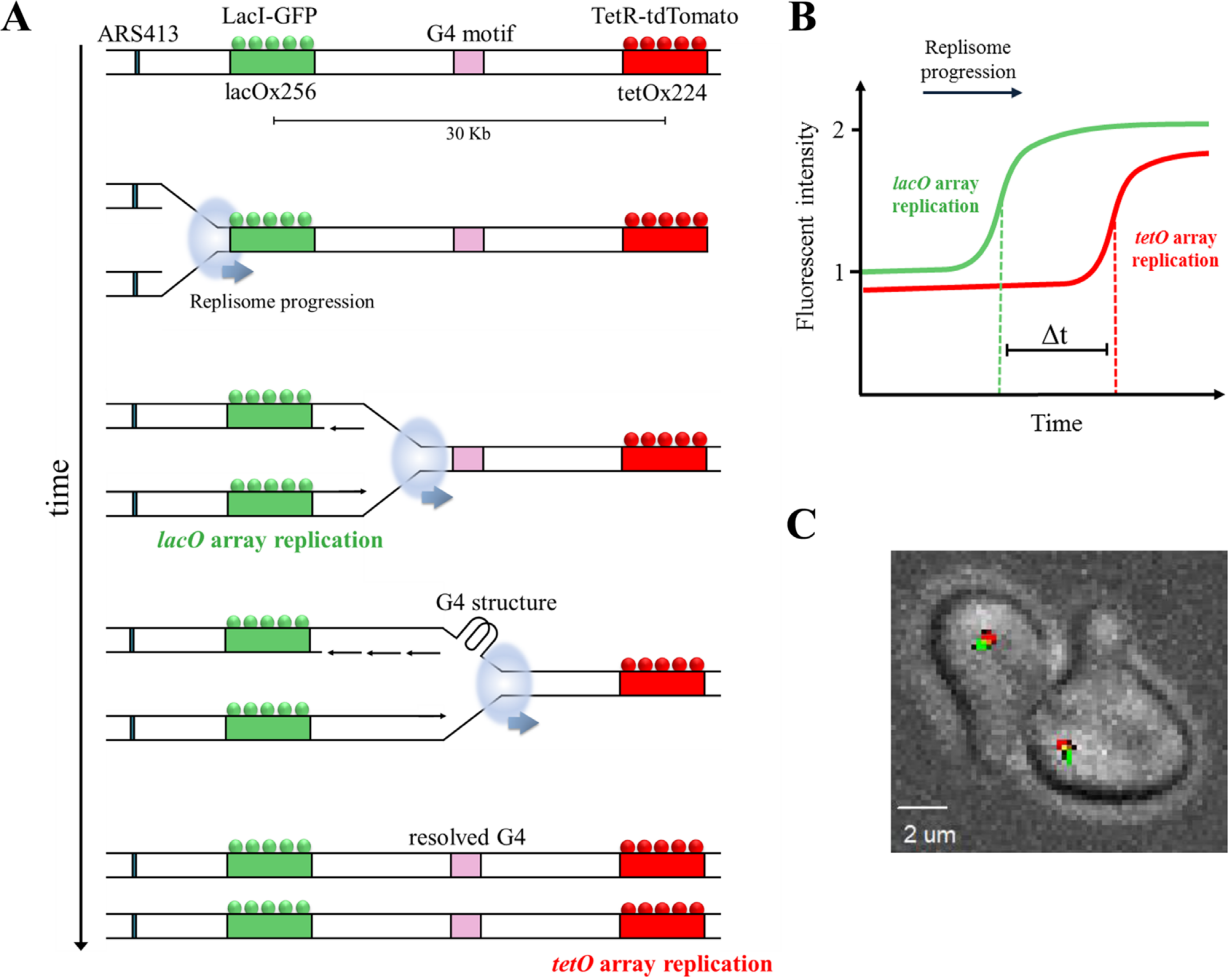
Measuring replication rates through G-quadruplex (G4) DNA secondary structures in single yeast cells. (**A**) Schematic illustration of the experimental design for real-time analysis of replication kinetics in live yeast cells. Strains are genetically engineered to contain *lacO* and *tetO* arrays adjacent to ARS413, located 20 Kb apart. Binding of lacI-GFP and tetR-tdTomato leads to green and red fluorescent foci, respectively. During DNA replication, array duplication leads to recruitment of additional lacI-GFP and tetR-tdTomato proteins leading to the doubling of fluorescence intensity. Using time-lapse confocal microscopy, the replication time of each locus is measured to calculate the replication rate of the DNA between the two loci. For simplicity, origin firing is shown only to the array direction. To measure replication through G4 structures, these G4 motifs are inserted between the arrays. (**B**) Schematic display of the increase in fluorescent intensity of the lacI-GFP and tetR-tdTomato foci due to *lacO* and *tetO* array duplication during DNA replication. The time delay between arrays replication (Δ*t*) is calculated using the mid-rise points of the GFP and tdTomato fluorescence intensities. Therefore, Δ*t* represents the replication time of ∼30 kb (addition of 10 kb to the 20 kb array spacing). (**C**) Image of single cells from the yeast strains used in this study.

Here, we examined the importance of Pif1 for DNA replication progression through G4 structures. We have utilized and modified our recently developed approach (19) for measuring the progression rates of single replication forks, as they replicate through G4 sequences, in living cells (Figure 1). We found that in the absence of Pif1 replication through lagging strand G4s is slowed down depending on the sequence and proximity of the G4s. In addition, we found that a canonical PIP box at the C-terminus of the helicase domain of Pif1 interacts with PCNA and that this interaction is essential for high replication rates through G4 structures *in vivo*. Our results provide direct *in vivo* evidence for the importance of Pif1 for replication through G4 sequences and suggest that Pif1 helicase activity is coordinated with replisome progression through Pif1–PCNA interaction.

## MATERIALS AND METHODS

### Microscopy and data analysis

All microscopy measurements were performed as described in (19) with slight modifications. Briefly, yeast cultures were grown to OD_600 nm_ = 0.1–0.3 in synthetic complete (SC) medium containing 4% glucose at 30°C and synchronized in G1 phase with 10 μg/ml α-factor (GenScript) for 2 hours. Cells were then immobilized in microscopy chambers (Ibidi) coated with 2 mg/ml Concanavalin A (Sigma Aldrich) and washed thoroughly with warm medium to remove α-factor before microscope observation. Cells were imaged on a Marianas spinning-disk confocal microscopy platform (3i), using an Evolve EM-CCD camera (Photometrics). 488 and 561 nm lasers were used for excitation of GFP and tdTomato, respectively. Imaging was performed at 1 min intervals and at a temperature of 28°C for 3–4 h, using an ×63 oil objective (NA = 1.4) in 3D (12 z-sections, 0.7 μm apart). Time-lapse data were collected with SlideBook (3i) and exported to Matlab for analysis using a custom-made package (‘DotQuant’) that identifies, tracks and quantifies the fluorescent foci in each cell (19). Statistical analysis of the results was performed using Monte Carlo resampling with 1 000 000 iterations.

### Strain generation

All strains were generated on the background of strain in which the *lacO* array was inserted at chrIV:332,960 and the *tetO* array at chrIV:352,560, to the right of ARS413 and with an expected mid-array distance of 30.6 kb. G4 motifs were inserted at genomic location chrIV:344119, 11.1 kb after the *lacO* array and 8.4 kb before the *tetO* array. The G4 sequence was integrated by replacing a *natMX* cassette inserted at the same genomic location, using a marker-free CRISPR/CAS9 mediated method (20). Briefly, yeast cells were transformed with donor DNA including the motifs flanked with homology to the genome together with the pCYn plasmid, that expresses the CAS9 endonuclease with gRNAs specific for the *natMX* cassette. This leads to cleavage of the cassette and high-efficiency replacement of the marker with donor DNA by homologous recombination. Donor DNA was generated by assembly of oligonucleotides that include the motifs (one G4 sequence for G4_(A or B)_ or two G4 sequences separated by a 96 bp random linker for G4_(A+B)_), flanked by 125 bp homology to the genome from each side.

*pif1Δ* was generated by replacing the *pif1* gene with a *hphMX* cassette. On this background *pif1-m2* and *pif1-PIPmut* strains were integrated into the native *PIF1* chromosomal location by a CRISPR/CAS9 mediated replacement of the cassette with the mutated alleles in a strain with G4_(A+B)_ background. Here, donor DNA was co-transformed with pCYh, with gRNA against the *hphMX* cassette. The mutated alleles were generated by PCR amplification of the WT *pif1* from the yeast genomic DNA with two primer pairs to generate two Pif1 fragments that overlap at the mutation site. Then, both PCR fragments were co-transformed to the yeast together with pCYh, leading to genomic integration of a full copy of the mutated gene via homologous recombination. All *pif1* mutants and G4 sequences were verified by DNA sequencing. The *pif1::pif1–6xFLAG* and *pif1-PIPmut::pif1-PIPmut-6xFLAG* strains were generated by amplifying a cassette containing 6xFLAG and *hphMX* marker from pHyg-AID*-6FLAG plasmid (21), with homology to the *pif1* terminator at the 5′ and *pif1* C-terminal at the 3′, followed by yeast transformation to WT and *pif1-PIPmut* strains. The plasmid pHyg-AID*-6FLAG was obtained from addgene (plasmid # 99519).

### ELISA

ELISA plates (Griener Microlon 96W) were incubated with 100 μl of 0.2 mg/ml streptavidin (Pierce) for 1 h, washed with PBS supplemented with 0.05% Tween-80 (PBST), and then 100 μl of 1 μM of biotinylated PIP peptides in PBS supplemented with 1% BSA (PBSF) were added to the plate for an additional hour. PBSF without peptide served as a negative control. The plates were then washed with PBST and blocked by incubation with 100 μl of PBS supplemented with 3% skim milk for 1 h. Following blocking, the plates were washed and incubated with 100 μl of 5 μM purified 6xhistidine-tagged PCNA and shaken for 1 h. Plates were then washed with PBST, incubated for 1 h with 100 μl mouse α-6xHis-tag antibodies (Santa-Cruz Biotechnology, 1:2000), washed, and incubated for 1 h with secondary horseradish peroxidase (HRP)-conjugated goat anti-mouse antibody (Jackson, 1:1000). Finally, 100 μl of HRP chromogenic TMB substrate solution (Dako) was added to each well. The reaction was stopped by the addition of 100 μl of 1 M sulfuric acid and recorded at 450 nm using a Tecan Infinite M200 plate reader.

### Western blot analysis of Pif1 expression

Yeast were grown in 50 ml YPD to OD_600_ 0.8, centrifuged, and lyzed using cell lytic solution (Sigma) supplemented with protease inhibitor cocktail (Sigma) and 7 mM DTT (Formedium), according to manufacturers' instructions. Lysates were concentrated with TCA (Acros) and loaded on to a 10% SDS-PAGE gel. Western blot analysis was performed using mouse α-FLAG (Sigma, 1:1000) or mouse α-Pgk1 (Invitrogen, 1:7000) primary antibodies and goat α-mouse HRP-conjugated (Jackson, 1:10 000) secondary antibody. All antibodies were diluted in PBS supplemented with 0.05% Tween-80 and 1% BSA.

### Purification of Pif1 and its PIP-box mutant variant

Pif1 variants were generated with standard site-directed mutagenesis protocols. Preliminary expression tests of a full-length Pif1 variant harboring mutations F760A, Y761A indicated that albeit the protein is expressed in *Escherichia coli*, it is poorly behaved during purification, consistent with recent observations (18). Therefore, we generated the F760A, Y761A mutations within a Pif1 construct that misses the first 237 aa (^Δ237^Pif1-PIPmut) and the proteins were overexpressed from a pET28b plasmid and purified as described (22). Wild-type Pol δ was purified as previously described (23), RFC, PCNA and RPA were a kind gift from Dr. Burgers (Washington University).

### ATPase, G-quadruplex stability and replication assays

The DNA-dependent ATPase activity of Pif1 and its mutant variant were determined spectrophotometrically using a NADH enzyme-coupled assay as previously reported (22). ATPase activity was measured at 20°C in Buffer A (10 mM Hepes pH 7.4, 100 mM NaCl, 8 mM Mg-Acetate, 1 mM DTT) as a function of the concentration of ssDNA (dT_60_) and at a constant concentration of 1 mM ATP. The stability of the G-quadruplexes was assayed by monitoring the change in absorbance at 295 nm, using a Varian Cary-100 spectrophotometer equipped with a Peltier-controlled cuvette holder. Oligonucleotides containing the sequences of G4_(A)_ and G4_(B)_ (Supplementary Table S3) were incubated in the indicated buffer for 3 min at 90°C, followed by slow cooling at room temperature. The G4-containing oligonucleotides, at a concentration of 3 μM, were incubated for 10 min at the starting temperature (14–16°C) followed by temperature increments of 2°C. Absorbance was measured after 3 min of incubation at each temperature increment. The normalized change in absorbance at 295 nm was fitted with a two-state model (24) using GraphPad Prism. Primer extension assays were performed by monitoring a fluorescently labeled primer annealed to a biotinylated template DNA strand in the presence of 100 mM KCl, as previously described (23). For primer sequences please see Supplementary Table S3. Briefly, replication assays were carried out in Buffer TM (20 mM Tris–HCl pH 7.8, 8 mM MgAc_2_, 1 mM DTT, 0.1 mg/ml BSA) with 100 mM KCl. RFC (20 nM) and PCNA (20 nM) were allowed to react with a single-biotinylated DNA substrate (20 nM) in presence of streptavidin (600 nM) and ATP (1 mM) for 2 min at 30°C, followed by the addition of Pol δ (20 nM) and dNTP mix (100 μM). RPA (40 nM) was added before Pol δ and incubated for 30 s at 30°C and Pif1 (40 nM) was added with Pol δ. The experiments in absence of PCNA were performed similarly but lacked ATP, RFC, PCNA, RPA and streptavidin. At the indicated times the reactions were stopped by the addition of 80 mM EDTA, 0.08% SDS and incubated at 55°C for 10 min. After addition of formamide (50% final), 20 mM EDTA, and 0.05% bromophenol blue, the samples were heated at 95°C for 2 min and analyzed on a 12% denaturing polyacrylamide gel, pre-run for 2 h in 1× TBE. The gels were scanned using a Typhoon 9400 Variable Mode Imager (GE Healthcare), monitoring the Cy3 fluorescence of the labeled primer, and quantified using ImageQuant.

## RESULTS

### Analysis of the site-specific replication rate of G4 containing sequence

We recently described an approach for measuring DNA replication rate at a specific genomic locus in single live *S. cerevisiae* cells (19). Here, we utilized this system to study replication through G4 secondary structures (Figure 1). The system is based on the replication of two different fluorescently marked arrays located near an early origin of replication. Specifically, we used the CRISPR/CAS9 technology (20) to insert arrays composed of multiple repeats of *lac* and *tet* operator sequences into the yeast genome, downstream to ARS413 and located 20 kb apart (Figure 1A). These operator repeats are bound by the fluorescent fusion proteins lacI-GFP and tetR-tdTomato, respectively, labeling both chromosomal loci as green (GFP) and red (tdTomato) dots under the microscope. When the arrays are replicated during S phase, the fluorescence intensity of each dot is doubled, since replication of the operators leads to the recruitment of more fusion proteins to each dot (Figure 1B). Using time-lapse confocal microscopy, we tracked the intensity of the fluorescence foci and identified when each locus is replicated (Figure 1C). Thus, the bacterial operator arrays serve as real-time *in situ* reporters of DNA replication, enabling the measurement of the replication rate at a specific genomic locus by measuring the time delay between the increase in green and red fluorescence intensity signal.

**Figure 2.**
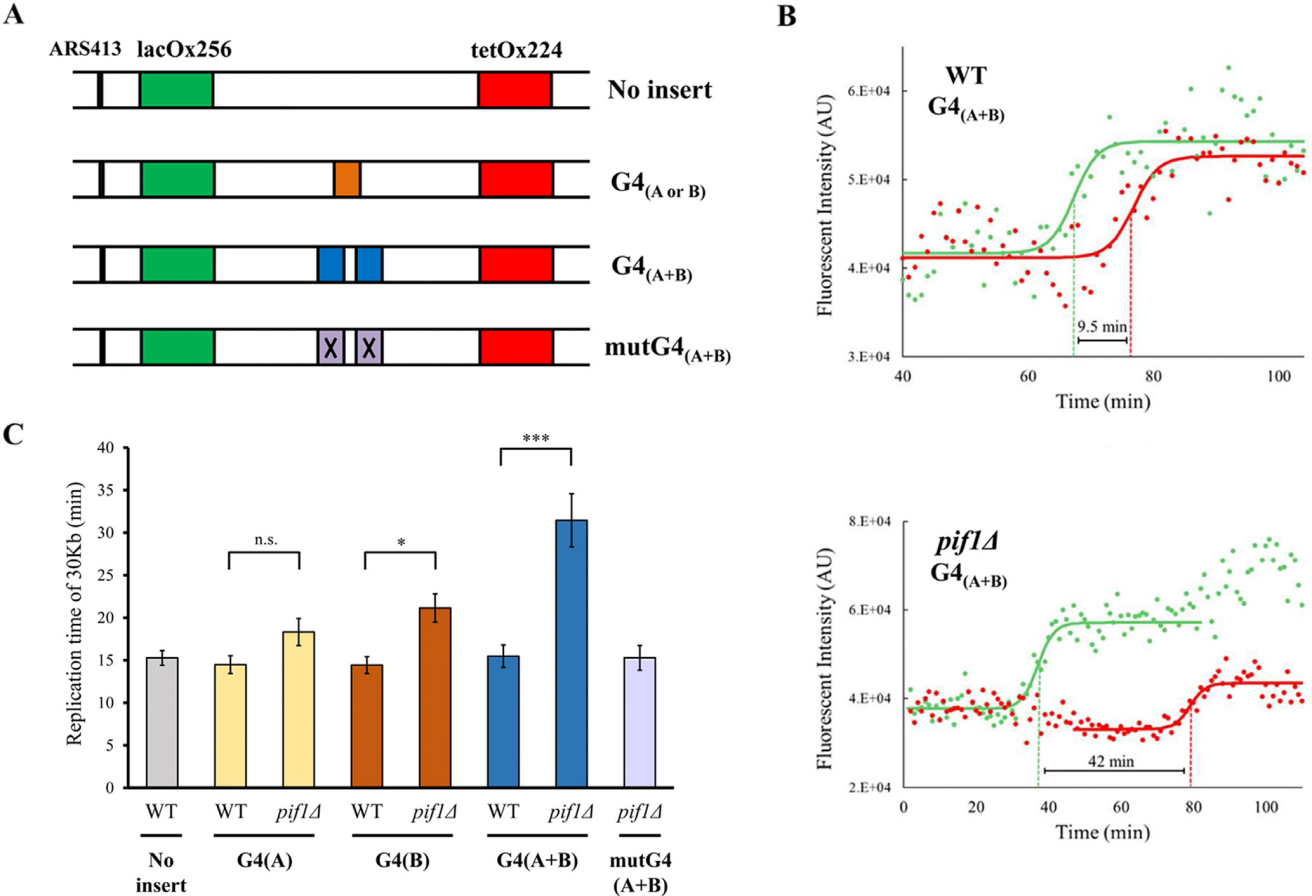
Pif1 is important for DNA replication through G4 structures located on the lagging strand. (**A**) Schematic illustration of the ARS413 chromosomal region containing G4 structures and a control strain. Replication was measured in strains with no insert between the arrays, either G4_(A)_ or G4_(B)_, G4_(A+B)_, or mutG4_(A+B)_ containing point mutations in the G4s that prevent the formation of the secondary structure. (**B**) Representative result of a single cell analysis for WT (top) and *pif1Δ* (bottom) strains with G4_(A+B)_ located on the lagging strand. Solid lines represent a fit of the data to a sigmoidal function, green and red mid-points are indicated by dashed lines. (**C**) Replication time of ∼30 Kb (distance between mid-*lacO* and mid*-tetO* arrays) for WT, *pif1Δ* in the different yeast strains containing the G4s between the arrays. Number of measurements (left to right) for WT (no insert), WT G4_(A)_, *pif1Δ* G4_(A)_, WT G4_(B)_, *pif1Δ* G4_(B)_, WT G4_(A+B)_, *pif1Δ* G4_(A+B)_, *pif1Δ* mutG4_(A+B)_ are *n* = 45, *n* = 25, *n* = 31, *n* = 24, *n* = 25, *n* = 22, *n* = 26, *n* = 25, respectively. All G4 elements were localized on the lagging strand. Error bars are ± SEM. Significance was determined by Monte Carlo resampling. *P< 0.05, ***P< 0.0005. Histograms of all cell measurements are shown in Supplementary Figure S2.

To study the effect of G4 structures on replication rate, two different G4 sequences were inserted between the operator arrays, either separately (G4_(A)_ or G4_(B)_) or in tandem (G4_(A+B)_) separated by a 96 bp linker (Figure 2A, see Supplementary Table S1 for DNA sequences). Previously, G4_(A)_ and G4_(B)_ sequences derived from the yeast chromosomes IX and IV, respectively, were shown to generate G4 secondary structures *in vitro* (4), were identified as Pif1 binding sites in yeast and were shown to induce genome instability in yeast in the absence of Pif1 (12). We found that these G4s display different stabilities *in vitro* (Supplementary Figure S1). As a control, we also inserted a mutated version of G4_(A+B)_ with point mutations that prevent the formation of secondary structures and *in vivo* recognition by Pif1 (12) (Figure 2A, mutG4_(A+B)_). Unless stated otherwise, all G4 sequences were inserted in lagging strand orientation.

### Pif1 is essential for fast replication through G4s

To examine the importance of Pif1 for replication through the G4 sequences integrated into the lagging strand, we measured replication rates in the yeast strains described above containing *PIF1* and *pif1Δ* (Figure 2). While replication rates were not affected by the insertion of one or two G4s in the WT strain, rates were slower when Pif1 was absent (Figure 2C, see Supplementary Figure S2 for replication times in individual yeast cells). Interestingly, we found that deletion of Pif1 does not have the same effect on replication through the G4_(A)_ and G4_(B)_ sequences. While replication through G4_(A)_ was only marginally slower in *pif1Δ* strain (*P* = 0.06), replication through G4_(B)_ was significantly slower when *PIF1* was deleted (*P* < 0.01, 1.5-fold decrease in replication rate). Moreover, a much stronger effect of *PIF1* deletion on replication rate was observed in a strain containing the tandem G4 sequences (G4_(A+B)_). We found that replication rate is slowed down by 2-fold in the G4_(A+B)_*pif1Δ* strain (*P* < 0.001), relative to the WT control strain, suggesting that tandem G4 sequences can act in an additive manner to slow down replication in the absence of Pif1. Importantly, replication through the mutated G4_(A+B)_ sequence in *pif1Δ* yeast exhibits WT-like rate, strongly suggesting that the change in replication rates in *pif1Δ* strains is dependent on the formation of G4 structures rather than the presence of G-rich sequence (Figure 2C). The change observed in replication rates between the different strains shows that Pif1 has a key role in allowing high replication rate through lagging strand G4s, especially in G4 rich genomic regions.

**Figure 3.**
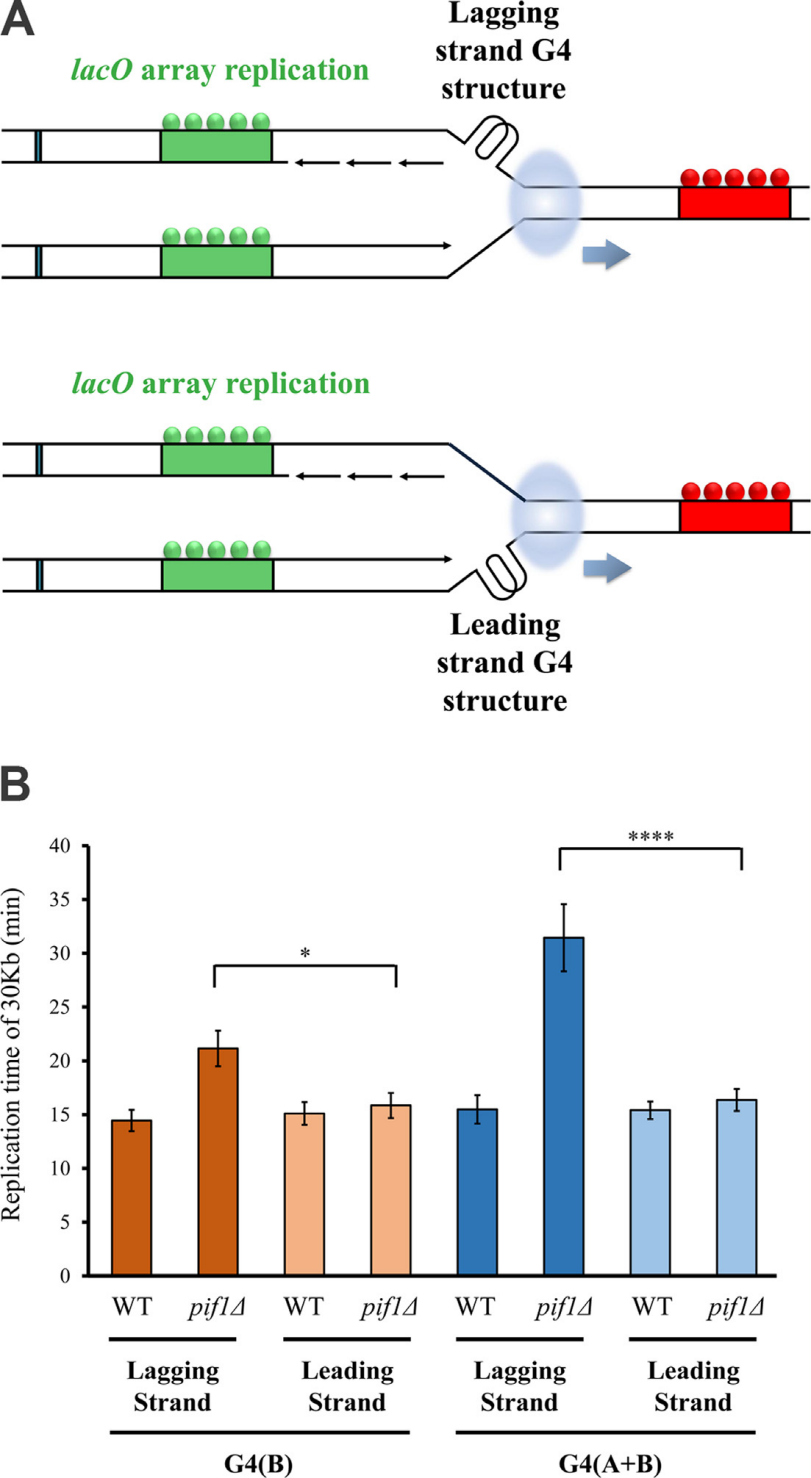
Pif1 is dispensable for high replication rate through G4 structures located on the leading strand. (**A**) Schematic illustration of G4 structures located on the lagging strand (top) or leading strand (bottom) with respect to replication fork progression in our system. (**B**) Replication time of ∼30 kb (distance between mid-*lacO* and mid*-tetO* arrays) for WT, *pif1Δ* containing G4_(B)_ or G4_(A+B)_ located on the lagging or leading strand. Replication times of *pif1Δ* strain containing G4_(B)_ or G4_(A+B)_ located on the leading strand are similar to WT strain. These times are significantly slower in *pif1Δ* strain containing G4_(B)_ or G4_(A+B)_ located on the lagging strand relative to the leading strand. Number of measurements (left to right) for WT G4_(B)_ (lagging), *pif1Δ* G4_(B)_ (lagging), WT G4_(B)_ (leading), *pif1Δ* G4_(B)_ (leading), WT G4_(A+B)_ (lagging), *pif1Δ* G4_(A+B)_ (lagging), WT G4_(A+B)_ (leading), *pif1Δ* G4_A+B_ (leading) are *n* = 24, *n* = 25, *n* = 31, *n* = 32, *n* = 22, *n* = 26, *n* = 25, *n* = 25, respectively. Error bars are ± SEM. Significance was determined by Monte Carlo resampling. *P< 0.05, ****P< 0.00005. Histograms of all cell measurements are shown in Supplementary Figure S2.

To examine whether the requirement of Pif1 for high replication rate through G4s is strand specific, we constructed additional WT and *pif1*-deleted strains in which G4_(B)_ or G4_(A+B)_ sequences were integrated into the leading strand (Figure 3). Interestingly, we found that replication rate in *pif1*-deleted strains containing G4_(B)_ or G4_(A+B)_, located on the leading strand, is dramatically higher relative to the respective strains containing G4_(B)_ or G4_(A+B)_ on the lagging strand and is similar to WT strains (Figure 3B). These results highlight that unwinding of these leading strand G4s is not Pif1 dependent and suggest that other helicases unwind these G4s enabling high replisome progression rates.

**Figure 4.**
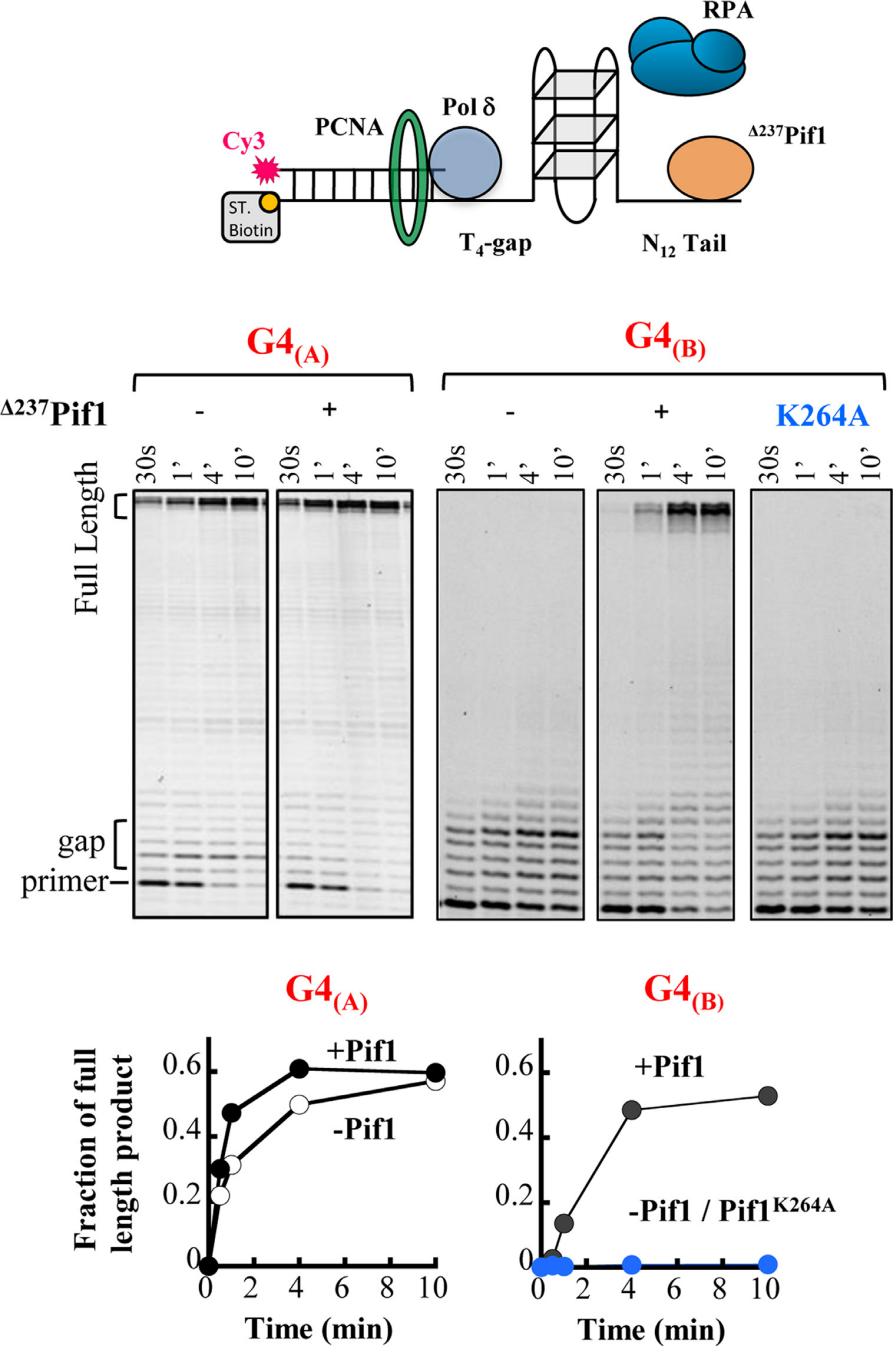
DNA primer extension activity of DNA polymerase δ on DNA templates that contain G4_(A)_ and G4_(B)_-DNA structures. The reactions were performed in the presence of PCNA, RPA, Pol δ and the absence or presence of ^Δ237^Pif1 (deletion of the first 237 aa of Pif1) or its ATPase deficient mutant. The lower panels are the quantitation of the gels above, reporting the fraction of the full-length products generated in the reaction.

To further probe the importance of Pif1 for replication through G4_(A)_ and G4_(B)_ sequences we examined DNA replication through the G4 sequences by DNA Polymerase delta (Pol δ) *in vitro* (Figure 4). Specifically, we used Pol δ primer extension assay through the G4 sequences in the presence of PCNA, Replication protein A (RPA), and with or without Pif1. For these experiments, we utilized a Pif1 version missing the first 237 amino acids (^Δ237^Pif1) that expresses to a much higher level in *E. coli* than the full length protein with no effect on Pif1 activity (22). In agreement with the *in vivo* data described above (Figure 2), we found that replication through the less stable G4_(A)_ is efficient even in the absence of Pif1, showing that this G4 sequence does not impose a significant barrier for Pol δ replication (Figure 4). In contrast, we found that replication through G4_(B)_ in the absence of Pif1 is completely inhibited even up to 10 min of incubation, consistent with this G4 forming a highly stable structure (Supplementary Figure S1). However, the addition of Pif1 allows Pol δ to replicate through the G4_(B)_ sequence, albeit at a slower rate relative to replication through the G4_(A)_ sequence (Figure 4). We found that DNA replication past the G4-DNA results from Pif1 unwinding of the G4 structure, as an ATPase deficient Pif1 did not stimulate the reaction (*pif1^K264A^*, Figure 4). These results further highlight the difference in G4 sequences acting as barriers for Pol δ replication and suggest that depending on their stability not all G4 sequences found in the yeast genome require Pif1 activity to allow optimal replication rate.

### Pif1 physically interacts with PCNA via a conserved PIP box

**Figure 5.**
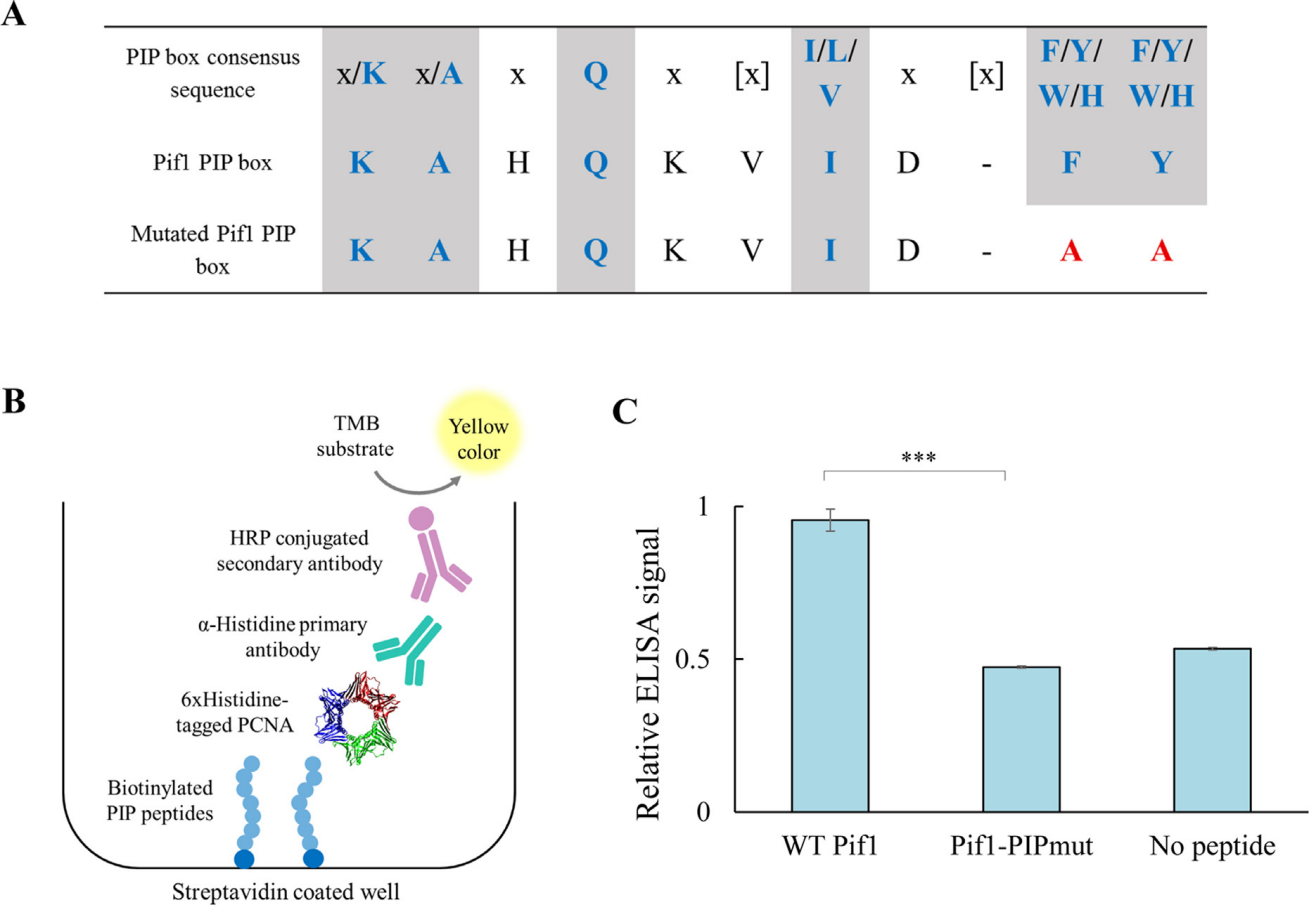
Pif1 physically interacts with PCNA via conserved PIP box. (**A**) Consensus PIP box (17) in comparison to identified PIP box in Pif1 and mutated PIP version (pif1-PIPmut). Consensus residues are marked in blue and mutations in red, [x] denotes spacing variations. Residues 752–761 in Pif1 are shown. (**B**) Schematic illustration of ELISA experimental setting for detecting the interaction between immobilized PIP peptides and PCNA. (**C**) Relative ELISA signal for PCNA–Pif1 PIP peptide interaction. Significance was determined by *t*-test. ***P< 0.0001. Error bars are ± SD, *n* = 3.

Since our data shows that Pif1 is important for high replication progression through G4s, we hypothesized that the activity of Pif1 at G4s may be mediated by PCNA. Recently, biochemical and structural data showed that Pif1 can interact with a non-canonical PIP box sequence located at the C-terminus of Pif1 (residues 817–823) to enhance Pol δ-mediated DNA synthesis (18). Additionally, physical interaction between PCNA and Pif1 has been observed in *S. cerevisiae* using a pull-down assay (25). Pif1 contains two short sequences located at the middle of the helicase domain (residues 372–380) and at the C-terminal of the helicase domain (residues 752–761) that resemble the known consensus for the PIP box (17). We decided to focus on the second PIP-like sequence (QKVIDFY) due to its location at the end of the helicase domain of the protein (Figure 5A). To test if PCNA can specifically recognize this PIP box region in Pif1, we used an enzyme-linked immunosorbent assay (ELISA) to examine the PCNA-PIP peptide interaction (Figure 5B, see Supplementary Table S2 for all peptide sequences). We have previously shown that this assay can be used to detect PCNA interactions with a variety of PIP peptides derived from different PCNA partners (26). We thus compared the binding of PCNA to short biotinylated peptides containing either the WT Pif1 PIP residues or a mutated version of the PIP box (pif1-PIPmut), where the F760 and Y761 were mutated to alanine (FY/AA, Figure 5A). We found that PCNA can bind the WT Pif1 PIP peptide, but not its mutated version (Figure 5C), showing that PCNA can specifically recognize this Pif1 PIP motif *in vitro*. As controls for this ELISA assay, we used WT and mutated forms of the PIP peptide derived from DNA polymerase η (Rad30), a known PCNA partner, and tested their interaction with PCNA (26,27) (Supplementary Figure S3). These results show that this canonical PIP region in Pif1 can contribute to the physical interaction between PCNA and Pif1, together with the other previously described non-canonical PIP region.

### Pif1 canonical PIP box is important for optimal replication through G4s

**Figure 6.**
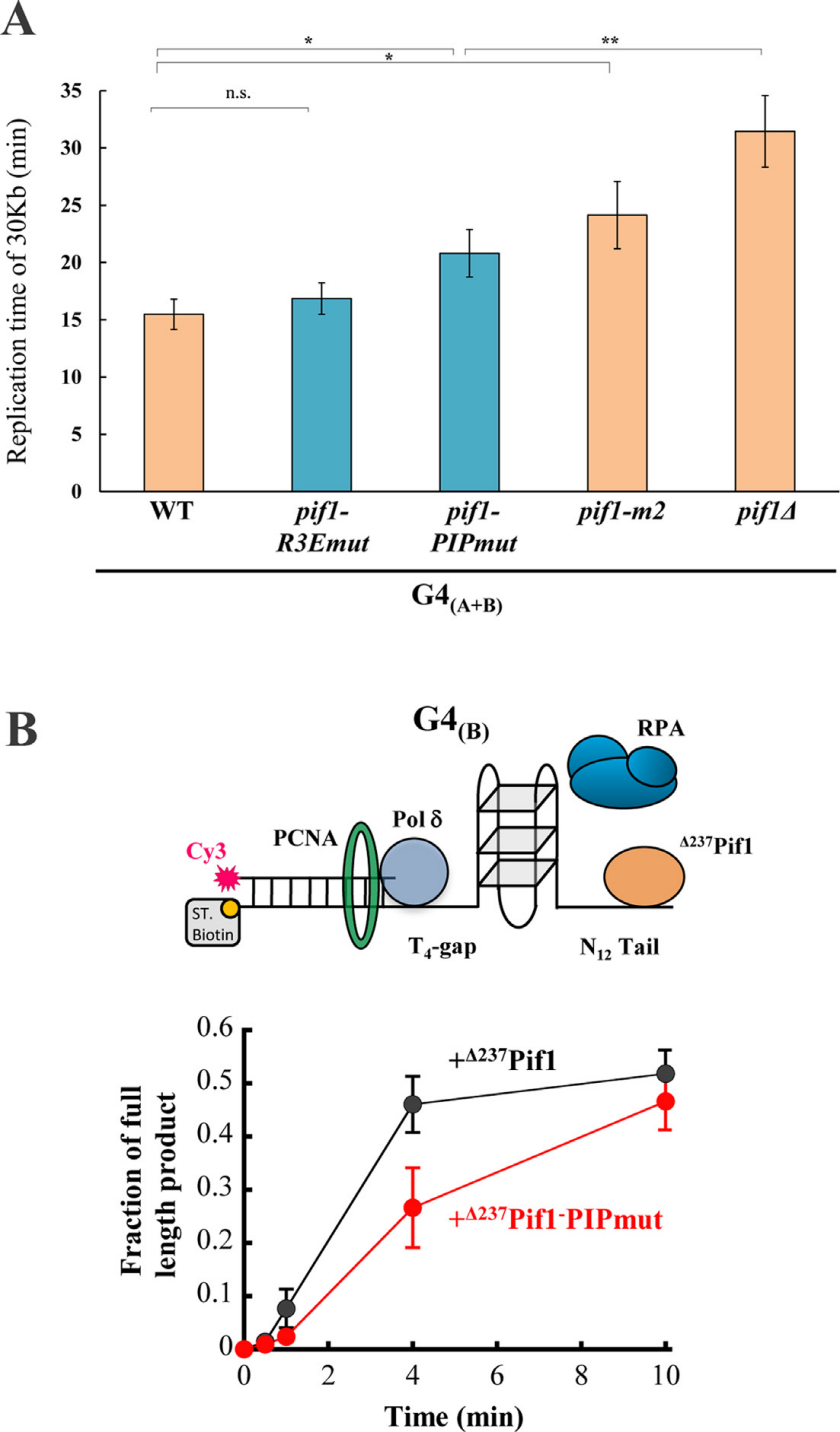
**(A)** Pif1–PCNA interaction is important for replication through G4 structures. Replication time of 30 Kb through G4_(A+B)_ located on the lagging strand in strains with *pif1-R3Emut* (*n* = 26)(18), *pif1-PIPmut* (*n* = 25) shown in blue and *pif1-*m2 (*n* = 26) in comparison to WT, *pif1Δ* (Figure 2C). Significance was determined by Monte Carlo resampling *P< 0.05, **P< 0.005. Error bars are ± SEM. (**B**) DNA primer extension activity of Pol δ past G4_(B)_ in the presence of the ^Δ237^Pif1 wild-type or its Pif1-PIPmut variant containing the F760A Y761A mutations. Standard deviations are from five independent experiments.

To examine the importance of the canonical PIP-mediated Pif1–PCNA interaction for replication through G4 secondary structures, we first generated a strain containing *pif1-PIPmut* by replacing the endogenous *PIF1* with the *pif1-PIPmut* containing the FY/AA mutations in the yeast genome, via a marker-less integration using the CRISPR/CAS9 system (20). This *pif1* mutation was generated on the background of a strain containing the G4_(A+B)_ sequence located on the lagging strand to allow measurements of replication through this sequence (Figure 2). Next, we used the cell-based assay described above (Figure 1) to examine how the FY/AA mutations in Pif1-PIPmut affect replication through the G4_(A+B)_ sequence. We found that the replication rate in this strain was significantly slower in comparison to WT (*P* < 0.05, 34% reduction in replication rate), but significantly faster than the *pif1Δ* strain (*P*<0.05, Figure 6A). The expression levels of WT Pif1 and *pif1-PIPmut* in the G4_(A+B)_ strains were validated by genomic tagging of the protein to 6xFLAG tag followed by western blot analysis (Supplementary Figure S4), while all replication rate measurements were performed with untagged *PIF1* variants. Next, we examined the growth rate of the *pif1-PIPmut* strain generated on a background of a strain that do not contain the G4s and observed a similar rate as the WT strain indicating that the PIP mutations in Pif1 do not reduce cell fitness (Supplementary Figure S5). To test the previously identified non-canonical PIP sequence (18) in our system, we measured replication through G4_(A+B)_ in a strain containing Pif1 with the mutated version of this PIP box (I817R, M820R, L821R, and R823E, *pif1-R3Emut*) (18). While these mutations were previously shown to impair the ability of Pif1 to enhance strand displacement synthesis by Pol δ *in vitro* and reduced BIR efficiency *in vivo* (18), they did not lead to a significant change in replication rate through the G4_(A+B)_ sequence (Figure 6A). We have also examined replication rate in strain containing the *pif1-m2* variant, a partial loss of function allele with near wild-type (WT) growth rates (28). Replication through G4_(A+B)_ was also slower in *pif1-m2* yeast (*P*<0.05, 1.6-fold decrease in replication rate), yet this effect was not as dramatic as in the absence of Pif1 (Figure 6A).

To further examine the importance of the canonical PIP box in Pif1 for Pol δ replication through G4 sequences, we used the *in vitro* assay described above (Figure 4). We first analyzed the effect of the F760A and Y761A mutations on the ATPase activity of Pif1 and its ability to unwind a duplex region downstream of a primer, thereby leading to an apparent stimulation of strand displacement DNA synthesis activity of Pol δ (22,29). Since the recombinant expression of the full length Pif1 containing the F760A and Y761A mutations was not possible due to protein aggregation (18), these mutations were generated in a Pif1 version missing the first 237 amino acids (^Δ237^Pif1-PIPmut) (Supplementary Figure S6A) and compared it to the deleted WT protein (^Δ237^Pif1) (22). We found that while this mutant exhibits a reduced ATPase activity (Supplementary Figure S6B), it is equally able to allow DNA synthesis activity by Pol δ into a duplex region as the WT Pif1 truncated version (Supplementary Figure S6C), indicating that the mutation does not affect helicase activity. We note that the apparent stimulation of strand-displacement DNA synthesis occurs in the absence of PCNA, indicating that interaction of Pif1 with PCNA is not strictly required (22). Importantly, ^Δ237^Pif1-PIPmut increased DNA synthesis past the strong G4_(B)_*in vitro*, albeit at a slightly slower rate compared to WT (Figure 6B). The ability of the Pif1-PIPmut to allow bypass of the G4 structure suggests either that additional Pif1–PCNA interaction sites are sufficient for stimulation of Pif1 activity, or that interaction of Pif1 with PCNA is not strictly required. To test the latter possibility, we performed the same reactions in the absence of PCNA (Supplementary Figure S7). We found that Pif1 increases DNA synthesis by Pol δ past this G4-DNA structure even in the absence of PCNA, confirming that this interaction is not strictly required for Pif1 G4 unwinding activity. These results are in agreement with the *in vivo* data showing a significantly smaller effect on replication slowdown in strain containing the Pif1-PIPmut relative to replication rate in the *pif1Δ* strain (Figure 6A). Taken together, our findings suggest that Pif1–PCNA interaction through the canonical PIP box is not essential for replication through G4s, however, it significantly increases replication rate through these regions possibly by coordinating and localizing Pif1 activity to the site of replication.

## DISCUSSION

In this study, we showed that Pif1 has a significant role in facilitating replication through G4 containing sequences. Previous works have shown contradicting results regarding the importance of Pif1 for replication fork progression through G4 sequences (12,14,15). The method used in this study enables the quantification of changes in replication fork progression at a single cell level and in a specific genomic locus (Figure 1). Utilizing this approach for measuring replication rates through G4 sequences allowed us to examine the extent of Pif1’s effect on replication through these regions even in the absence of DNA damaging agents such as HU (Figure 2). Our results suggest that Pif1 has a key role in replication fork progression through lagging strand G4 secondary structures, however, this role is dependent on the G4 sequence and distribution. Pif1 deletion caused a significant replication slowdown only through G4_(B)_ derived from chromosome IV, but not through G4_(A)_ derived from chromosome IX. This correlates well with differences in loop length and thermal stability of the two G4_(A)_ or G4_(B)_ structures (Supplementary Figure S1 and Table S1) allowing DNA replication through some G4s even in the absence of Pif1 (Figure 4). Our results are in good agreement with previous examination of various G4-forming sequences both *in vitro* and *in vivo* that showed that short G4 loop length leads to increased thermal stability and decreased *in vivo* genomic stability in yeast (30). In contrast, examination of the epigenetic instability of the BU-1 locus in REV1-deficient DT40 cells due to different G4 sequences, has shown that the G4 effect is dependent on being located on the leading strand but is independent of its *in vitro* thermal stability (31).

Our replication assay performed on individual cells allows examining cell to cell variations and thus obtaining more detailed analysis of replication through G4s compared to experiments performed on cell populations. Interestingly, we found high variability of replication times in *pif1*-deleted or mutated strains containing lagging strand G4s, including some cells exhibiting replication times similar to WT cells (Supplementary Figure S2). A possible explanation for cells exhibiting WT replication times is the melting of G4 structures in these cells. Our biophysical characterization of the G4s shows sharp denaturation curves that are best fitted to a two-state model in which G4s can adopt either a fully folded or fully unfolded state (Supplementary Figure S1 and Materials and Methods section). If G4s occasionally adopt an unfolded state *in vivo*, replication times in this subpopulation should be similar to WT, as observed in *pif1*-deleted strains that do not contain structured G4s (e.g. mutG4_(A+B)_, Figure 2C). Thus, our reported population-averaged replication times in G4-containing strains (e.g. Figure 2C) are the lowest estimate, while replication times in individual cells that actually contain a folded G4 structure can be much higher (Supplementary Figure S2).

Our finding that G4_(B)_ and G4_(A+B)_ located on the leading strand do not slow down replication in a *pif1*-deleted strain (Figure 3) suggests that other helicases may unwind leading strand G4s. Previously, DNA polymerase ϵ, which mediates leading strand replication, was shown to be physically associated with the CMG helicase (32) raising the possibility that the CMG helicase itself may be sufficient for G4 unwinding in the absence of Pif1. The effect of G4s located on the lagging versus leading strand remains a point of debate. A previous study (12) has shown that direct repeat recombination levels in *pif1*-deleted cells are not dependent on G4 strand orientation. However, the authors have also shown that lagging strand G4s can lead to increased level of mutations under HU conditions, suggesting that G4s in this orientation pose a higher stress to cells (12). In an additional study by Lopes *et al.* (13), human G4 *CEB1* tandem array was integrated into WT and *pif1*-deleted strains near *ARS305* in a lagging or leading strand orientation. The authors found a dramatic increase in destabilization of the CEB1 motif and replication pausing in the leading strand relative to the lagging strand orientation in *pif1*-deleted background. The differences between our study (Figure 3) and the previous study (13), may stem from differences in the number of repeats and sequence of the G4s, assay for characterization and genomic location.

A more dramatic effect on replication was obtained by inserting both G4 structures in tandem (G4_(A+B)_), indicating the synergistic effect of two G4 regions and the high importance of Pif1 in these cases. Regions containing more than one G4 motif were found in repetitive regions of the genome, suggesting that Pif1 may have a crucial role in enabling the replication of these regions. For example, the human CEB1 minisatellite forms stable G4 structures *in vitro* and causes G4-dependent genomic instability when introduced to Pif1 depleted *S. cerevisiae* (11,13). Additionally, the G-rich strand of various telomeres can form G4 structures *in vitro*, and there is evidence for their formation *in vivo* in *Stylonychia lemnae* and in human cultured cells telomeres (8). G4 motifs in the same proximity of the tandem G4s used in this study, or closer, can be found in conserved regions in the *S. cerevisiae* genome in over 30 cases, excluding telomeric DNA (4).

Additionally, we found that Pif1 contains a canonical PIP box at the C-terminus of the Pif1 helicase domain that interacts with PCNA (Figure 5). In the absence of this interaction, replication through G4s is slower, yet this change in replication rate is not as dramatic as the effect of deletion of *PIF1* (Figure 6A). This result suggests that the activity of Pif1 at G4s is not completely dependent on its interaction with PCNA through the PIP box described above. This result agrees with our biochemical data showing that Pif1 can function in the absence of PCNA in mediating Pol δ replication through G4 containing sequences (Supplementary Figure S7); however, Pif1-PIPmut leads to slower Pol δ DNA synthesis through G4 relative to the WT (Figure 6B). Thus, the Pif1 interaction with PCNA through the canonical PIP box described above enhances Pol δ mediated replication through G4 regions possibly by coordinating Pif1 activity with fork progression. The PIP box discovered in this work differs from the non-canonical PIP box recently identified (18). However, mutating this PIP sequence in our G4_(A+B)_ strain did not show a significant effect on replication through G4s (*pif1-R3Emut*, Figure 6A). It was previously shown by a pulldown experiments that residual PCNA binding by a Pif1 fragment that does not include the non-canonical PIP box still exists (18). Thus, it is possible that more than one sequence in Pif1 can mediate PCNA interaction and that these different sequences mediate distinct Pif1 functions including break-induced replication (18) and replication through G4 sequences as described here. Previously, multiple PIP boxes were also identified in Pol δ and were shown to be important for Pol δ *in vitro* and *in vivo* activity (33).

It has been recently proposed that Pif1 has a post-replicative role in resolving G4s before mitosis rather than unwinding these structures during DNA replication (14). In this scenario, replication forks bypass the G4 structure leaving nicks and gaps that are later filled, without resolving them during replication (12). The importance of Pif1–PCNA interaction for replication through G4s as described here suggests that Pif1 is critical for G4 processing during replication. In accordance with this model, we did not detect elongation of the G2 cell cycle phase in the *pif1*-deleted strain that usually occurs when post-replication repair is activated (Supplementary Figure S8). We propose that Pif1 interacts with PCNA to enable replication through G4s and that this interaction is important to prevent replication fork stalling at G4 motifs. This may be followed by additional Pif1 binding and unwinding to G4s in late S/G2 phase, for final validation that all G4s are resolved before mitosis (12). This model is consistent with the known rise of Pif1 cellular abundance in late S/G2 phase (34).

A role for accessory helicases in aiding replication across G-quadruplexes is not unique to yeast Pif1. Formation of telomeric G4-DNA has been proposed to impair telomere replication, thereby leading to telomere fragility (35–37). Loss of RTEL helicase was shown to lead to an increase in fragile telomeres (38,39), providing the first indication that RTEL has a role in aiding replication across these difficult to replicate structures. Interestingly, RTEL interacts with PCNA and mutations of its C-terminal PIP-motif led to an increase in fragile telomeres as well, pointing to a crucial role for this interaction in RTEL function at G4-DNA, similar to our observation with yeast Pif1.

In summary, the slowdown of replication at lagging strand G4-containing sequences and the importance of Pif1–PCNA interaction for replication fork progression through these structures highlight the functions of Pif1 during replication for optimal fork progression through these G4 sequences. In this case, PCNA may be important for the localization of Pif1 at G4 containing sequences during replication enabling the correct timing of G4 unwinding during this process.

## Supplementary Material

Supplementary Data
