# Supplementary material for: Pif1 is essential for efficient replisome progression through lagging strand G-quadruplex DNA secondary structures
Source: Nucleic Acids Res. 2018 Nov 5;46(22):11847–57. doi: 10.1093/nar/gky1065 (PMC6294490; doi:10.1093/nar/gky1065)
Supplement: Supplementary Data [file gky1065_supplemental_files.docx]

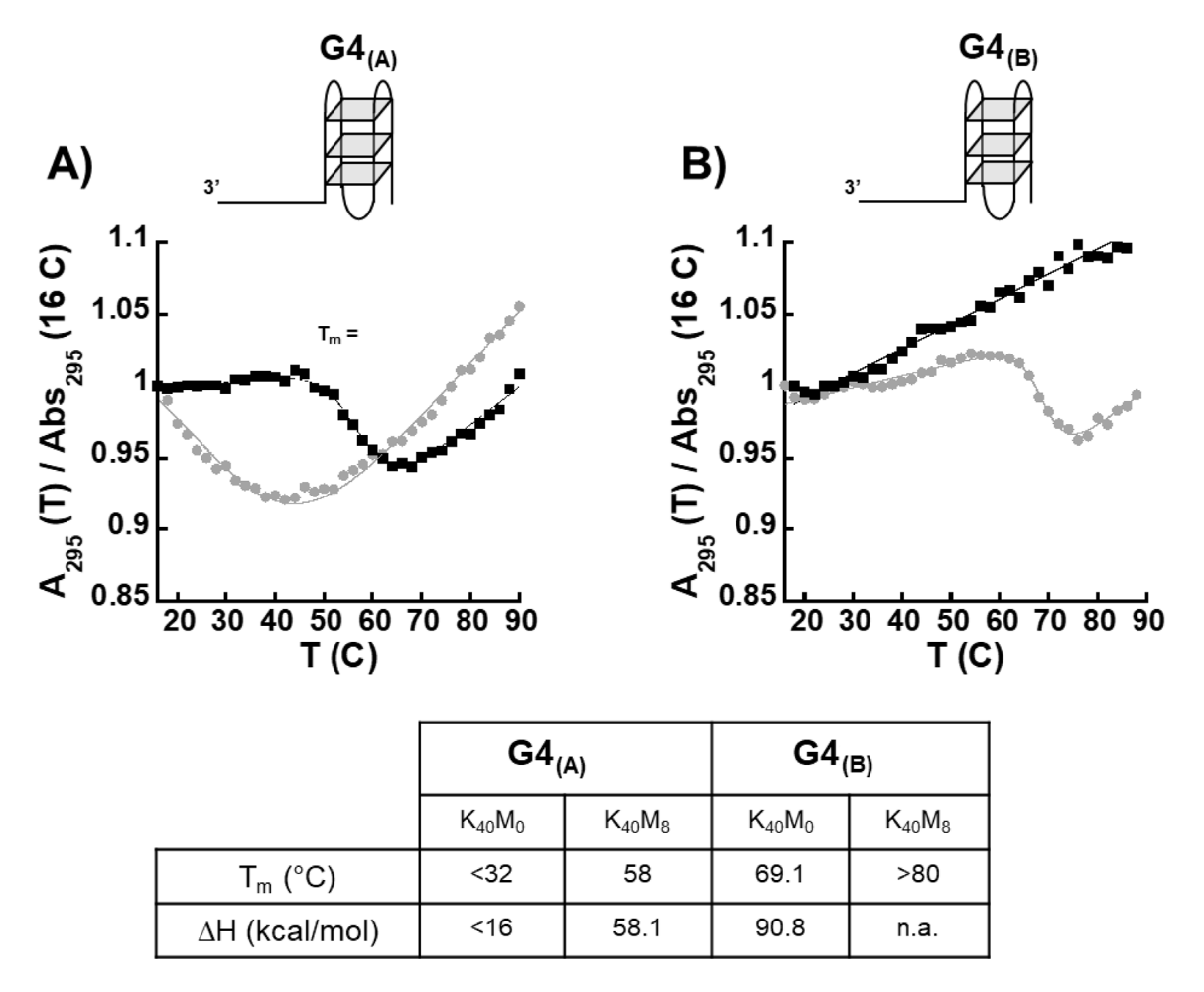


**Figure S1: G4_(A)_ forms a less stable G-quadruplex than G4_(B)_. A)** UV melting of 3 μM G4_(A)_ in 20 mM HEPES pH7.4 and 40 mM KCl (gray) or 40 mM KCl and 8 mM MgAc_2_ (black). The solid lines are fitting to a two-state model with parameters in the table below. **B)** Same as in A) but for G4_(B)_.


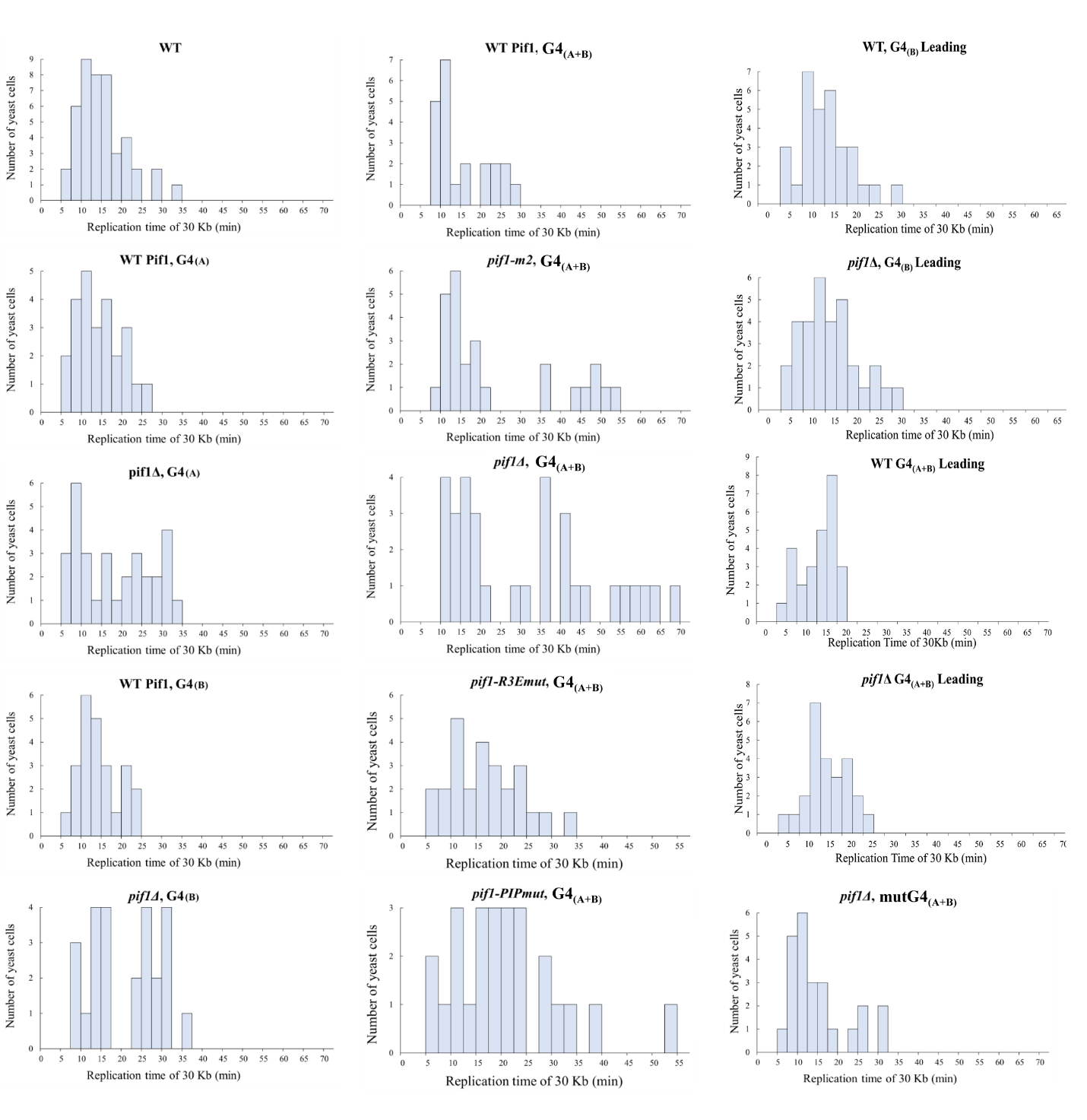


**Figure S2**: Distribution of replication times of 30 Kb (min) within each yeast strain population described in this study (**Fig. 2-3** and **Fig. 6**).


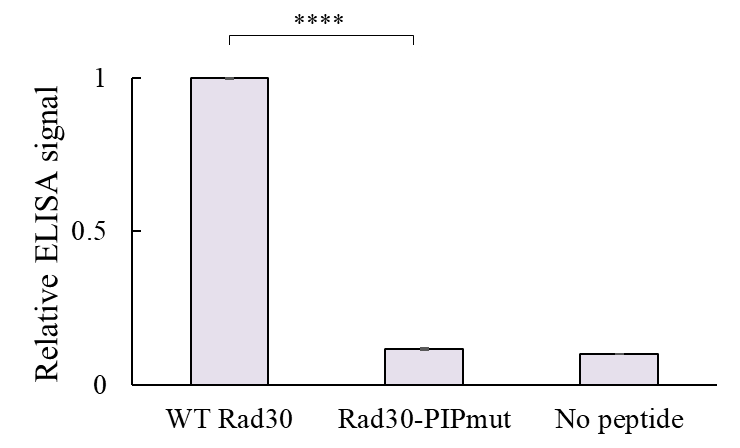


**Figure S3**: Relative ELISA signal for PCNA-Rad30 PIP peptide interaction. Significance was determined by t-test. ****p<0.0001. Error bars are ± SD, n=3.

**
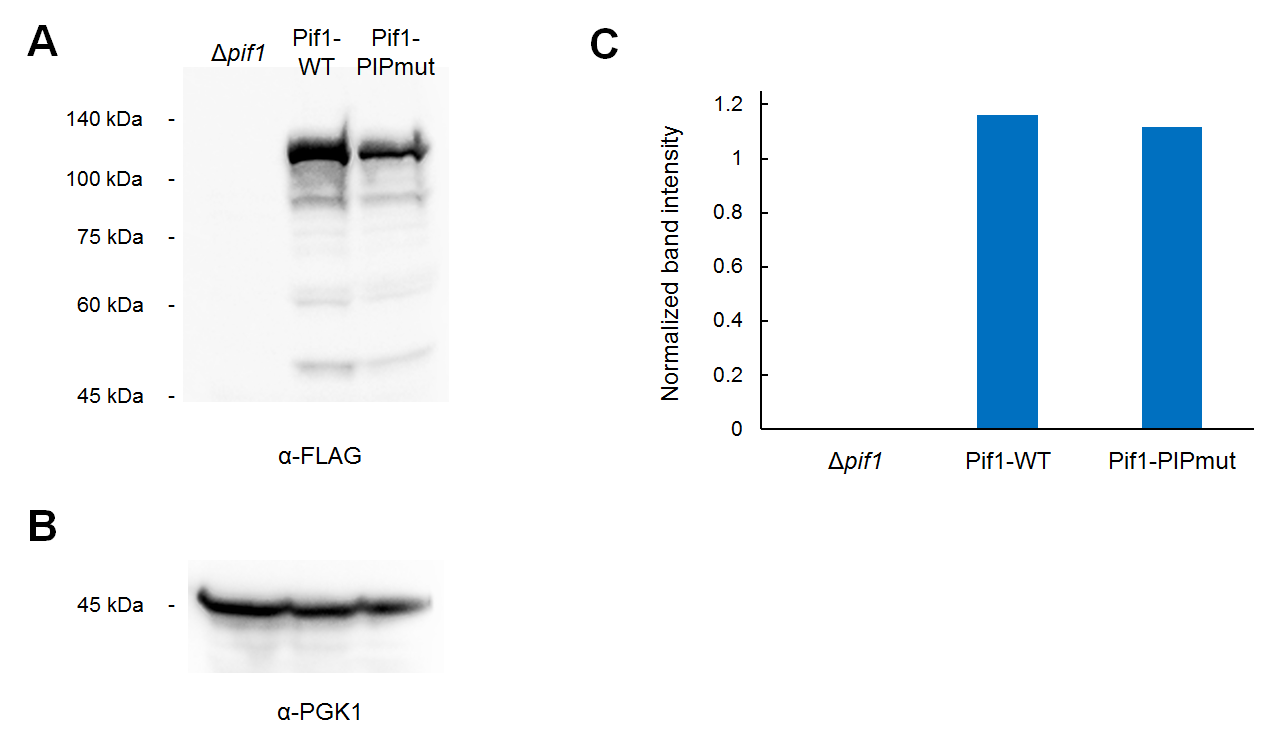
**

**Figure S4:** Expression of WT and mutant Pif1 showing no significant difference in expression between the proteins. Both Pif1-WT and Pif1-PIPmut were C-terminally tagged with 6xFLAG, in the endogenous PIF1 locus. (**A, B**) Whole cell extracts of Δ*pif1*, Pif1-6xFLAG and Pif1-PIPmut-6xFLAG were loaded onto 10% SDS-PAGE gel, transferred to nitrocellulose and probed with mouse α-FLAG (**A**) or mouse α-PGK1 (**B**, loading control) primary antibodies respectively, and goat α-mouse HRP-conjugated secondary antibody. The expected molecular weights are: Pif1-6xFLAG – 106kDa, PGK1 – 45kDa. (**C**) Quantification of Pif1-6xFLAG band intensities, normalized according to PGK1 band intensities to correct for differences in loading.


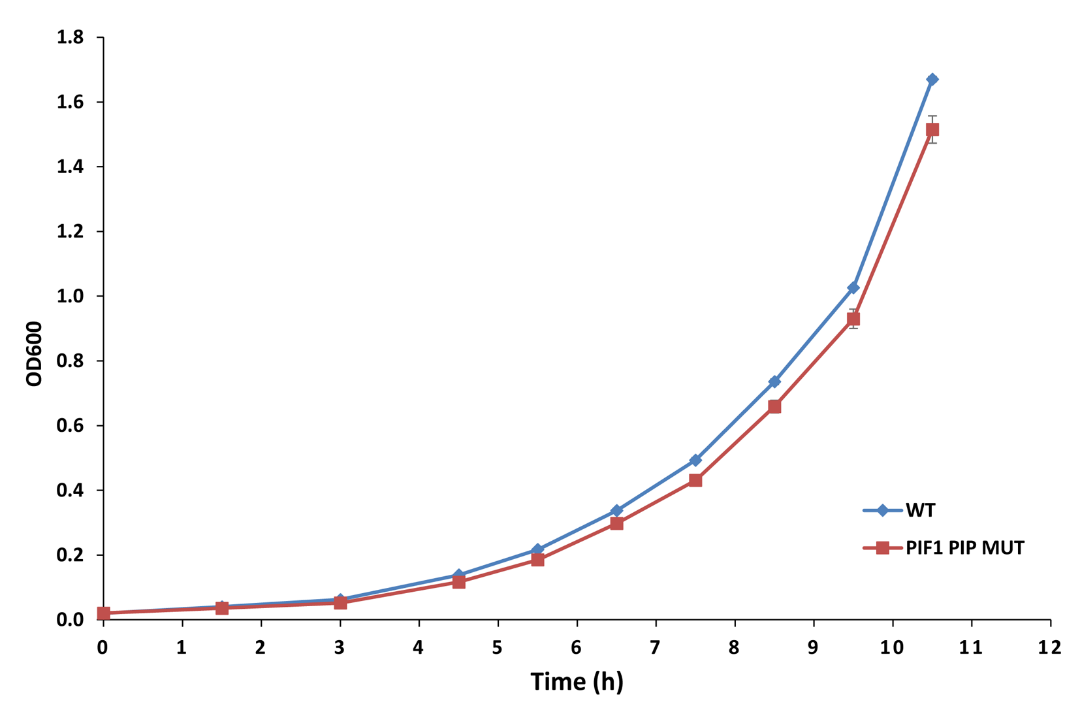


**Figure S5**: Growth curve analysis of untagged WT and pif1-PIPmut strains indicating no difference in growth rate between the strains. Cells were grown in a synthetic complete (SC) media and all measurements were performed in triplicates. To calculate the growth rate, we used a linear regression following logarithmic transformation of the OD_t_=OD_0_·2^t/τ^ equation to obtain Log_2_ OD_t_ = Log_2_OD_0_ + t/τ; the slope value of the linear fit is 1/τ. The generation time (doubling time (τ)) for the WT was found to be 1.68 ± 0.02 and for the pif1-PIPmut 1.65 ± 0.02.


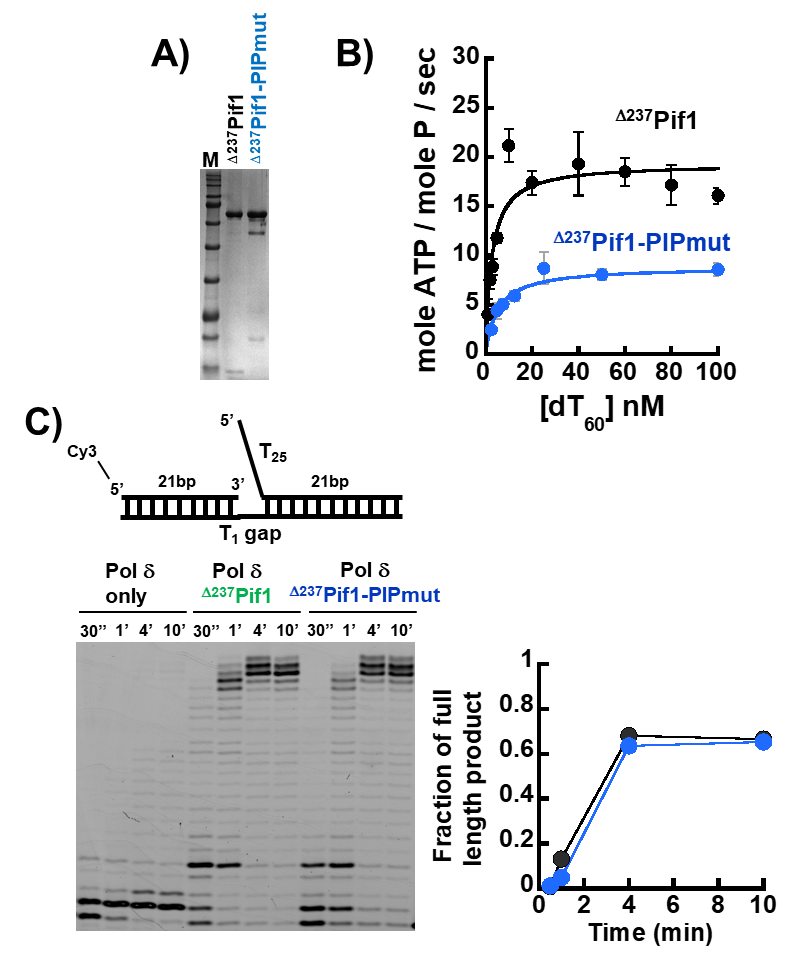


**Figure S6: A)** SDS-PAGE of a ^Δ237^Pif1 construct missing the first 237 aa and its variant ^Δ237^Pif1-PIPmut containing the F760A and Y761A mutations. **B)** DNA dependent ATPase activity of the two Pif1 constructs. **C)** Strand-displacement DNA synthesis of Pol δ alone and in the presence of either construct of Pif1.


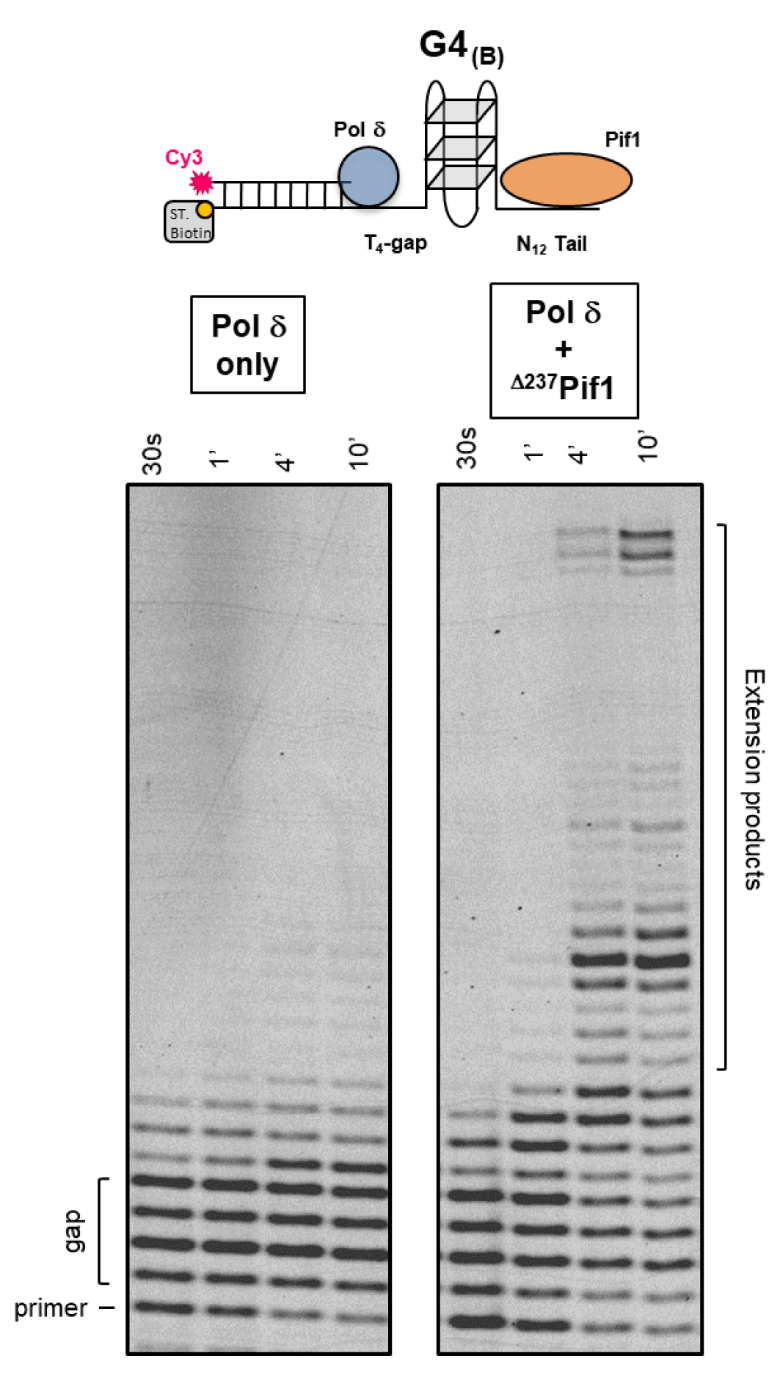


**Figure S7:** DNA primer extension activity of Pol δ (in the absence of PCNA) past the G4-DNA from G4_(B)_, in the absence and presence of Pif1.


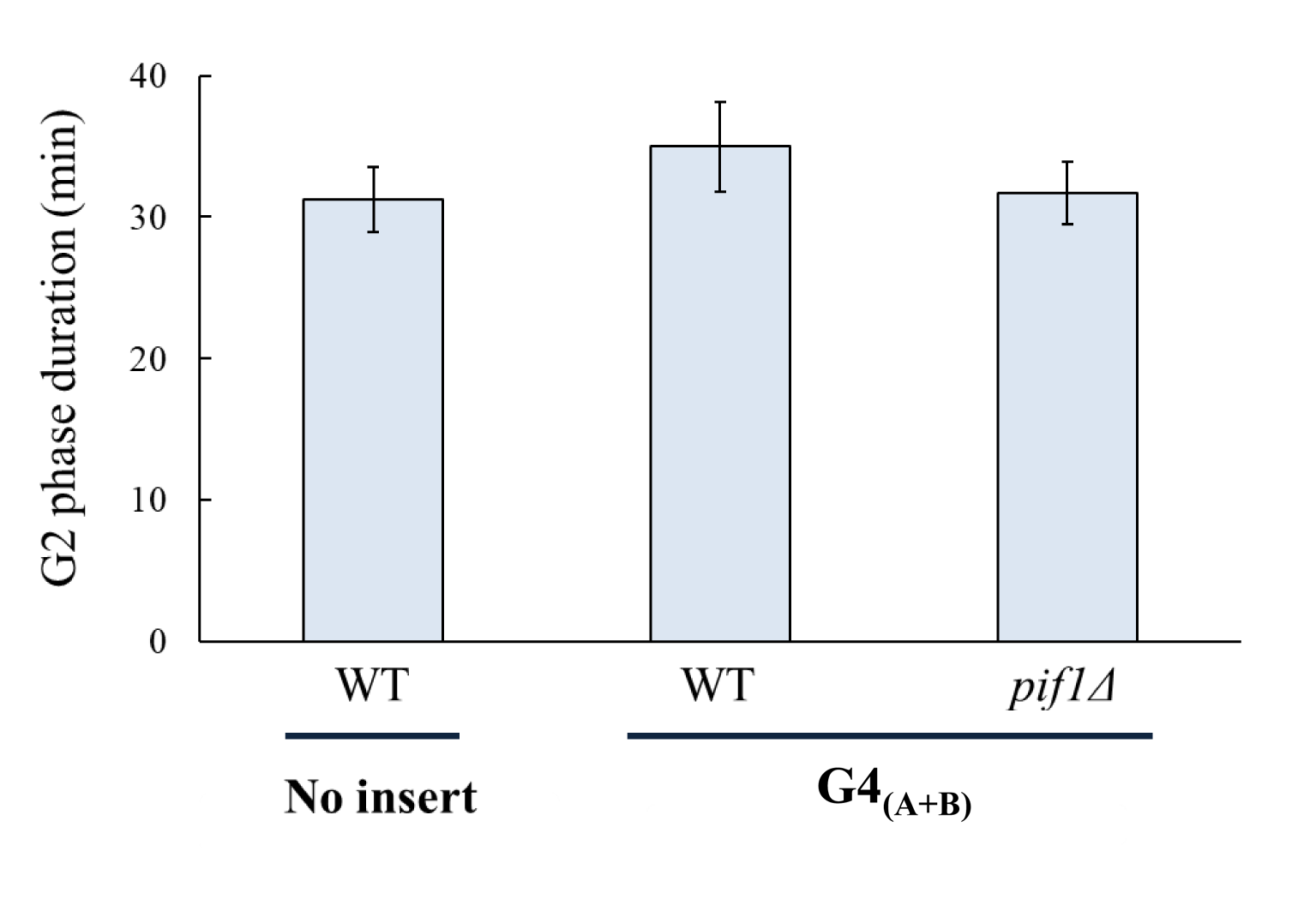


**Figure S8**: Length of G2 cell cycle phase. G2 phase duration was calculated by measuring the time interval between replication of *tetO* array (signifying the end of S phase) and mitosis. There is no significant change in G2 phase duration between the strains. Significance was determined by Monte Carlo resampling. Error bars are ± SEM (n=43, n=21, n=26, respectively).

**Table S1:** G4 sequences used for *in vivo* experiments (5’-3’)

| G4_(A)_ | GGGTACGGTGGGTAATAAGGGAAGGTATCGGG |
| --- | --- |
| G4_(B)_ | GGGGAGGGGAAGGGGAGGGG |
| G4_(A+B)_  (G4s are underlined) | GGGTACGGTGGGTAATAAGGGAAGGTATCGGGTTAGATCCCAGTCGAATGGATTAATCAAACAGATCTGTAGCCGGAGAGGCATACCCCCTGCGACACTTTACGAAGGCATCTGCAAAAATCATAACTGGGGAGGGGAAGGGGAGGGG |
| mutG4_(A+B)_  (mutated G4s are underlined) | GCGTACGGTGGGTAATAACGCAAGGTATCGCGTTAGATCCCAGTCGAATGGATTAATCAAACAGATCTGTAGCCGGAGAGGCATACCCCCTGCGACACTTTACGAAGGCATCTGCAAAAATCATAACTGGCGAGCGGAAGGGGAGCGG |

**Table S2:** PIP peptides used in this study

| WT Pif1 | Biotin-RTRIKAHQKVIDFYLTLSS-NH2 |
| --- | --- |
| Pif1-PIPmut | Biotin-RTRIKAHQKVIDAALTLSS-NH2 |
| WT Rad30 | Biotin-QKKQVTSSKNILSFFTRKK-NH2 |
| Rad30-PIPmut | Biotin-QKKQVTSAKNALSAATRKK-NH2 |

**Table S3:** Sequences of DNA substrates for *in vitro* experiments

|  | **DNA Sequence (5’-3’)** |
| --- | --- |
| Primer | Cy3-CCGCCGCGGAACTTATTAGTG |
| G4_Chr IX  (G4_(A)_) | ACGTCATTGGTCGGGTACGGTGGGTAATAAGGGAAGGTATCGGGTTTTCACTAATAAGTTCCGCGGCGG-Bio |
| G4_Chr IV (G4_(B)_) | ACGTCATTGGTCGGGGAGGGGAAGGGGAGGGGTTTTCACTAATAAGTTCCGCGGCGG-Bio |
| StdDis_top strand | TTTTTTTTTTTTTTTTTTTTTTTTTCGCTATATTACCCGGAGTACA-Bio |
| StdDis_template | TGTACTCCGGGTAATATAGCGTCACTAATAAGTTCCGCGGCGG-Bio |
